# Genetic and environmental determinants of bone quality: a cross-sectional analysis of the Hungarian Twin Registry

**DOI:** 10.1007/s11357-024-01265-2

**Published:** 2024-07-02

**Authors:** Szilvia Mészáros, Márton Piroska, Tamás Leel-Őssy, Ádám Domonkos Tárnoki, Dávid László Tárnoki, Zsófia Jokkel, Helga Szabó, Éva Hosszú, Emőke Csupor, Réka Kollár, Árpád Kézdi, Ádám G. Tabák, Csaba Horváth

**Affiliations:** 1 Department of Internal Medicine and Oncology, Faculty of Medicine, Semmelweis University, Budapest, Hungary; 2 Medical Imaging Centre, Faculty of Medicine, Semmelweis University, Budapest, Hungary; 3 Hungarian Twin Registry, Budapest, Hungary; 4 2nd Department of Pediatrics, Faculty of Medicine, Semmelweis University, Budapest, Hungary; 5 Health Service, Buda Castle Local Authorities, Budapest, Hungary; 6 Department of Public Health, Faculty of Medicine, Semmelweis University, Budapest, Hungary; 7 Károly Rácz Conservative Medicine Division, Doctoral College, Semmelweis University, Budapest, Hungary; 8 UCL Brain Sciences, University College London, London, UK

**Keywords:** Bone quality, Trabecular bone score, Hungarian Twin Registry

## Abstract

There is abundant evidence that bone mineral content is highly heritable, while the heritability of bone quality (i.e. trabecular bone score [TBS] and quantitative ultrasound index [QUI]) is rarely investigated. We aimed to disentangle the role of genetic, shared and unique environmental factors on TBS and QUI among Hungarian twins. Our study includes 82 twin (48 monozygotic, 33 same-sex dizygotic) pairs from the Hungarian Twin Registry. TBS was determined by DXA, QUI by calcaneal bone ultrasound. To estimate the genetic and environmental effects, we utilized ACE-variance decomposition. For the unadjusted model of TBS, an AE model provided the best fit with > 80% additive genetic heritability. Adjustment for age, sex, BMI and smoking status improved model fit with 48.0% of total variance explained by independent variables. Furthermore, there was a strong dominant genetic effect (73.7%). In contrast, unadjusted and adjusted models for QUI showed an AE structure. Adjustments improved model fit and 25.7% of the total variance was explained by independent variables. Altogether 70–90% of the variance in QUI was related to additive genetic influences. We found a strong genetic heritability of bone quality in unadjusted models. Half of the variance of TBS was explained by age, sex and BMI. Furthermore, the adjusted model suggested that the genetic component of TBS could be dominant or an epistasis could be present. In contrast, independent variables explained only a quarter of the variance of QUI and the additive heritability explained more than half of all the variance.

## Introduction

Osteoporosis is characterized by low bone mass (i.e. quantity) and altered bone microarchitecture (i.e. quality, or non-mass bone property), resulting in decreased bone strength and increased fragility [1].

Bone mineral density (BMD) is accepted as a major, but not the only determinant of fragility. Fragility is also influenced by bone quality that is the microarchitecture of bone as well as the material properties that are not measured by dual-energy X-ray absorptiometry (DXA). Bone quality is a complex term that includes several bone properties partly determining bone resistance to fracture [2]. Bone quality is usually divided into geometric and material properties. Geometric properties include the macroscopic geometry of the whole bone and the microscopic architecture within the bone tissue. Material properties encompass the composition and arrangement of the microstructural constituents (collagen and mineral), as well as microdamage and microstructural discontinuities (such as microporosity and lamellar boundaries) [3]. Bone quality has a direct effect on the mechanical properties (for example the elasticity) of bone and determines its strength and elasticity.

Bone quality is difficult to test in clinical practice. While various procedures are available for research (for example high-resolution peripheral computer tomography [HR-pQCT]), only two measures (trabecular bone score [TBS] and quantitative ultrasound index [QUI]) are frequently determined in routine care. TBS provides indirect information about structure, while quantitative ultrasound (QUS) gives a composite of bone mass and aspects of quality.

QUI provides information on bone mechanical and structural properties [4]. It is a composite of broadband ultrasound attenuation (BUA) and speed of sound (SOS). BUA reflects bone mass and some aspects of microstructure, like trabecular separation and connectivity. SOS mainly reflects trabecular separation and elasticity [5] while less influenced by bone mass. QUS variables reflect different material properties of bone, such as elastic modulus and compressive strength, which is influenced by its density, architecture and elasticity [5]. This complicated horizon can play a limiting role in the use of QUS; however, this is the only method providing any information on bone quality in daily practice.

Over the last decade, new technologies to measure bone quality were developed using DXA results. The most successful of these is TBS that represents a new texture parameter coming from pixel gray-level variations in DXA images at the lumbar spine. TBS represents bone microstructure and is an index of bone fragility. Accumulating evidence suggests that TBS could improve fracture risk assessment [6–8] as it is related to fracture risk independently of BMD, age [9] and FRAX [10].

There is abundant evidence from mutigenerational [11–14] and twin studies [15–22] that DXA measures of bone mineral content are highly heritable (50–80%) although common variants (mean allele frequencies > 5%) only explain a small proportion (10–20%) of the variation of BMD measures. There is also some evidence for the high heritability of bone geometry [11, 23]. In contrast, much less is known about the heritability of bone quality measures. There is a multigenerational analysis from the Framingham Offspring study on HR-pQCT measures, as well as on SOS and BUA from twin and multigenerational family studies [14, 16, 24, 25]. To the best of our knowledge, only one multigenerational family study investigated the heritability of TBS [26] and one that of QUI [14] leaving considerable uncertainty in our knowledge about the heritability of these bone quality measures.

Given this shortage of information, we aimed to disentangle the relative role of genetic, shared and unique environmental factors on bone quality using two non-mass bone surrogates (TBS and QUS) among male and female Hungarian twins.

## Materials and methods

### Setting and participants

**Fig. 1:**
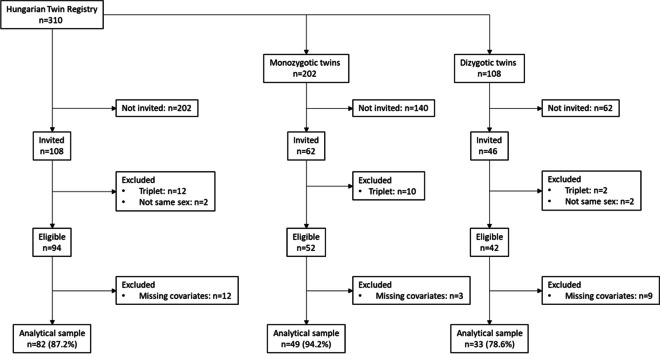
Flow chart of study participants. Numbers are given for twin pairs/triplets

The present study is embedded in the voluntary Hungarian Twin Registry that included 310 twin pairs (65% monozygotic [MZ], 35% dizygotic [DZ], 70% women, mean age 44 ± 16 years) at the time of recruitment [27]. A convenience sample of 108 non-pregnant twin pairs were invited for a clinical assessment that included a brief physical examination and a questionnaire as well as a bone DXA and QUS scan in 2019. After the exclusion of triplets and non-same-sex twins, 94 twin pairs were eligible. After the exclusion of those with missing co-variates, the final analytical sample included 82 twin pairs (87.2% of those eligible; 48 monozygotic [MZ], 33 same-sex dizygotic [DZ] twin pairs) (Fig. 1).

The present study was approved by the National Scientific and Ethics Committee (ETT TUKEB 189–4/2014) and was carried out according to the principles stated in the Declaration of Helsinki. All subjects provided written informed consent.

### Measures

Zygosity was assigned using a multiple-choice self-reported questionnaire and latent class analysis in accordance with previous recommendations [28].

Age, sex and smoking habit (current smoker yes/no) were derived from the self-reported questionnaire.

Weight and height were measured in light clothing on a digital scale to the nearest 0.1 kg and to the nearest 1 cm before the bone scan. Body mass index (BMI) was calculated as weight (kg)/height^2^ (m). Usual physical activity (PA) was estimated by the International Physical Activity Questionnaire (IPAQ), converted to metabolic equivalent task (MET) minutes per week and reported as inactive (< 600 METmins), minimally active (600–1499 METmins) and active (≥ 1500 METmins) [29].

Bone mineral density and TBS for each participant were determined by dual x-ray absorptiometry (Discovery WI, Hologic Inc, USA) by the same trained operator and in accordance to the manufacturer’s instructions. BMD was determined at the lumbar spine (L1–L4), femoral neck, total hip and the radius. TBS of the L1–L4 vertebral bodies was calculated using the TBS iNsight® software (ver. 3.1, MediMaps Group, Geneva, Switzerland) installed on the DXA machine. The coefficient of variation for repeated measures of TBS was 1.49%.

Heel BMD and QUS measurement on the calcaneus was performed in a sitting position using a waterless device (Sahara Clinical Bone Sonometer, HOLOGIC, Bedford, MA, USA). We estimated QUI using the measured speed of sound (SOS) and broadband ultrasound attenuation (BUA) using the following equations: QUI = 0.41 × (BUA + SOS) − 571.

The coefficient of variation (CV) for QUI and estimated heel BMD was 1.89 and 2.19%, respectively.

### Statistical analysis

Descriptive statistics are given as means with standard errors for continuous variables and counts and percentages for categorical variables by zygosity. The groups of monozygotic and dizygotic twins were compared with two-sample *t*-tests and *χ*^2^ tests for age and sex with twin pairs as the unit of observation, as age and sex did not differed within twin pairs. All other variables were compared using generalized estimating equations (GEE) with logistic link for categorical and identity link for continuous variables to take into account the multilevel structure of the data.

To estimate the genetic and environmental effects on bone quality measures, we utilized a classical twin design (CTD). Twins are born and raised at the same time; MZ twins have the same genetic background, while DZ twins share on average 50% of their genes. Based on this theoretical background, it is possible to decompose the variance of an outcome into the following components: additive genetic influence (A), shared environmental influence (C) and unique environmental influence (the error term of the decomposition—E). This type of analysis is called ACE-variance decomposition [30].

To be able to estimate the A and C components, the following assumptions are required. First, the genetic effects should be additive, such that the effects of different genes are independent. Second, the genetic and environmental components are also additive; thus, there are no correlations or interactions between genes and the environment. The third is the equal environments assumption (EEA), which states that MZ twins are similarly treated by their environment to DZ twins. The fourth is that spouses mate randomly.

We estimated the genetic and environmental effects using two sets of models. First, we estimated ACE-variance decomposition using the linear multilevel mixed-effects parameterization developed by Rabe-Hesketh et al. [31] without any adjustment (*Model 0*). Then, we extended the model by including the following explanatory variables: age, sex, body mass index and ever smoker status (*Model 1*). Both outcomes as well as age and BMI were normalized for these analyses; thus, 1 unit increase in TBS, QUI, age and BMI corresponds to one SD change (0.13 for TBS, 22 for QUI, 14.4 years for age and 5 kg/m^2^ for BMI).

To select the final model (separately for model 0 and model 1), first we checked whether the C component could be removed from the model based on its point estimate (< 5%), *p*-value (> 0.05) or by comparing the fit of the ACE and AE models using likelihood ratio test (*p* > 0.05). If the C component could be removed and the ratio of the correlation coefficients within monozygotic to within dizygotic twins was over 2, we investigated whether the addition of a dominant (D) genetic component would improve the fit of the model again using likelihood ratio test. This way, the final models could include the following variance components: ACE or AE or ADE.

We report for each model the fixed effects for age, sex, BMI and ever smoker status with their respective 95% confidence intervals (CI), as well as the percentage of variance partitioned (with its 95% CI) to each of the A/C/D/E components over the sum of the variance partitioned to these components. For graphical representation, we partitioned the overall variance to the percentage explained by the independent variables (age, sex, BMI, ever smoker status) and each of the A/C/D/E components.

Descriptive analyses were computed with SPSS version 28 for Windows, and ACE models were computed with Stata version 15.1 using acelong.ado [32]. Two-sided *p* values were used with an alpha level of 0.05 for statistical significance.

## Results

### Baseline characteristics by zygosity

Baseline characteristics of the study population by zygosity are presented in Table 1. While MZ twins were approximately 6 years younger compared to DZ twins (*p* = 0.05), we found no difference between the groups in the other baseline measures. Over 70% of the participants were women, a quarter of them were current or prior smoker. MZ and DZ participants had similar weight, height and BMI with a mean value in the overweight category. The distribution of the usual physical activity was similar in the two groups with > 80% of the participants engaging in at least some exercise. BMD and qualitative bone measures were within the normal range and did not differ significantly between the groups, although all point estimates were lower in the DZ group probably related to their older age (Table 1).

**Table 1:** Baseline characteristics of study participants by zygosity

	Monozygotic twins		Dizygotic twins		*p*
*Variable*	Mean/*n*	SE/%	Mean/*n*	SE/%	
*n*	98		66		
Age* (years)	53.4	2.2	59.5	2.1	0.05
Male*	18	36.7%	7	21.2%	0.151
*Ever smoker*	22	22.4%	18	27.3%	0.559
*Height (m)*	1.67	0.01	1.65	0.01	0.378
*Weight (kg)*	72.7	1.7	74.7	1.6	0.482
*BMI (kg/m*^*2*^*)*	26	0.5	27.5	0.7	0.134
*Physical activity (METmins)*					0.447
< *600*	11	11.2%	12	18.2%	
*600–2999*	50	51.0%	35	53.0%	
≥ *3000*	36	36.7%	19	28.8%	
*Lumbar BMD*	1.001	0.016	0.980	0.021	0.547
*Femoral neck BMD*	0.801	0.016	0.775	0.016	0.356
*Radius BMD*	0.658	0.010	0.626	0.011	0.094
*Trabecular bone score*	1.401	0.014	1.384	0.015	0.492
*Calcaneus BMD*	0.514	0.013	0.499	0.015	0.547
*Quantitative ultrasound index*	93.5	2.1	90.9	2.4	0.538

All other *p*-values are based on generalized estimating equations

*n* refers to the number of individuals included in an examination

*BMI* body mass index, *MET* metabolic equivalent of task, *BMD* bone mineral density, *SE* standard error

^*^Unit of observation—twin pair, *p*-values are based on two-sample *t*-test and *χ*^2^ test

### Heritability of TBS

The final unadjusted model (*model 0*) with TBS as the outcome included no shared environmental effect (C) and although the point estimate of the ratio of the MZ to DZ correlations was over 2, the ADE model showed a poorer fit compared to the ACE model, leading to an AE model suggesting an > 80% additive genetic heritability in trabecular bone score (Table 2).

**Table 2:** ACE decomposition model characteristics with trabecular bone score (TBS) as the outcome

	Model 0										Model 1						
	ACE model			ADE model			AE model				ACE model				ADE model		
	Estimate	95% CI	*p*	Estimate	95% CI	*p*	Estimate	95% CI	*p*	Estimate		95% CI	*p*	Estimate		95% CI	*p*
*Male*	NA			NA			NA			0.04		− 0.24; 0.33	0.77	0.05		− 0.22; 0.33	0.71
*Age (/SD)*	NA			NA			NA			− 0.61		− 0.75; − 0.48	< 0,0001	− 0.61		− 0.75; − 0.48	< 0.0001
*BMI (/SD)*	NA			NA			NA			− 0.08		− 0.2; 0.03	0.15	− 0.1		− 0.21; 0.02	0.1
*Eversmoker*	NA			NA			NA			0.06		− 0.18; 0.29	0.57	0.06		− 0.16; 0.28	0.58
*A*	82.5	67.7; 91.4	0.016	85.4	85.4; 85.4	< 0.0001	85.5	81.3; 88.8	< 0.0001	71.7		63.2; 79.1	< 0.0001	0		0; 100	1
*D*	NA			0	0; 0									71.5		73.7; 73.7	< 0.0001
*C*	3	0; 100	0.93	NA	0; 100	0.93				0		0; 100	1				
*E*	14.5	10.2; 20.1	< 0.0001	14.6	10.4; 20	< 0.0001	14.5	10.3; 20.1	< 0.0001	28.2		20.3; 37.7	< 0.0001	28.5		19.6; 34.4	< 0.0001
*MZr*	0.87	0.73; 1.01	< 0.0001	0.87	0.73; 1.01	< 0.0001	0.87	0.73; 1.01	< 0.0001	0.74		0.55; 0.93	< 0.0001	0.74		0.55; 0.93	< 0.0001
*DZr*	0.4	0.07; 0.72	0.016	0.4	0.07; 0.72	0.016	0.4	0.07; 0.72	0.016	− 0.12		− 0.47; 0.23	0.5	− 0.12		− 0.47; 0.23	0.5
* − 2LL*	357.842			357.852			357.852			292.06				287.02			
*p*							0.92 (vs. ACE)			< 0.0001 (vs. Model 0)							

*95% CI* 95% confidence interval, *A* additive genetic variance, *BMI* body mass index, *C* common or shared environmental variance, *D* dominant genetic variance, *DZr* intraclass correlation coefficient for dizygotic twins DZ twins, *E* unique environmental variance, *LL* log-likelihood, *MZr* intraclass correlation coefficient for monozygotic twins

*p*-values are given for the comparison of the − 2LL values between different models

*Model 0*: ACE-variance decomposition without adjustment

*Model 1*: Model 0 + age, sex, body mass index and ever smoker status (model 1)

Both outcomes as well as age and BMI were normalized for these analyses; thus, 1 unit increase in TBS, QUI, age and BMI corresponds to one SD change (0.13 for TBS, 14.4 years for age and 5 kg/m^2^ for BMI)

**Fig. 2:**
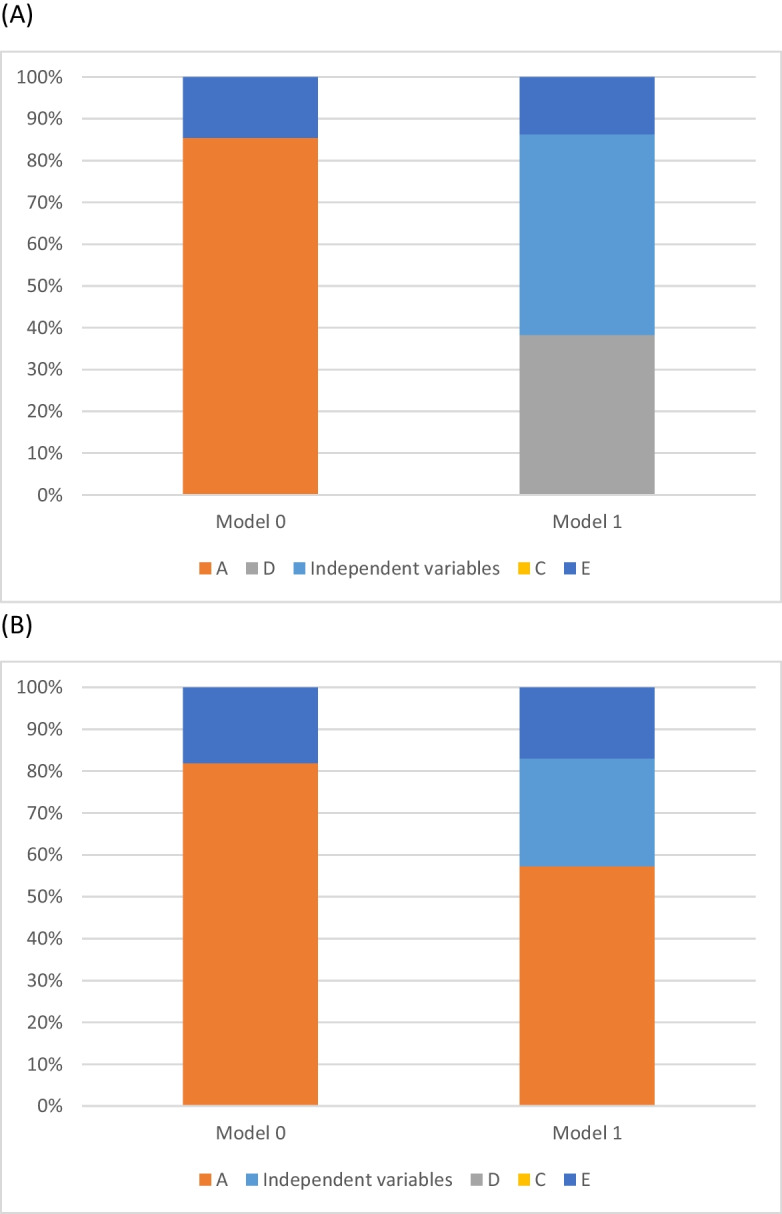
Total variance and standardized variance components for trabecular bone score (TBS—**A**) and quantitative ultrasound index (QUI—**B**). *Model 0*: ACE-variance decomposition without adjustment. *Model 1*: Model 0 + age, sex, body mass index, and ever smoker status (Model 1). Variance components (% of total variance) are shown in each bar. A, additive genetic variance; C, common or shared environmental variance; D, dominant genetic variance; E, unique environmental variance; independent variables—age, sex and body mass index

Adjustment for age, sex, BMI and ever smoker status hugely improved the model fit (*model 1*) with almost half of total variance (48.0%) explained by independent variables, most strongly by age. Given that the twins were examined at the same time, there was no heterogeneity in age within twin pairs; thus, we cannot investigate whether aging is genetically determined or is part of the shared environment. Furthermore, after age adjustment, the correlation within DZ twins became non-significant, suggesting a strong dominant genetic inheritance. This was further supported by the fact that the ADE model had a better fit compared to the ACE model. According to the final adjusted model, there was a strong dominant genetic effect (73.7%) and the rest of the explained variance was related to non-shared environmental factors (Table 2, Fig. 2A).

### Heritability of QUI

In contrast, the unadjusted (*model 0*) and the adjusted (*model 1*) were more consistent for QUI with both an AE structure. While the point estimate for the shared environment (C) was relatively large, its omission from the final model did not worsen its fit significantly.

Adjustment for age, sex, BMI and ever smoker status significantly improved the model fit approximately and a quarter (25.7%) of the total variance was explained by these independent variables. Altogether 70 to 90% of the variance in QUI was related to additive genetic influences, while 10 to 30% to non-shared environmental factors with overlapping confidence intervals for *model 0* and *model 1* (Table 3, Fig. 2B).

**Table 3:** ACE decomposition model characteristics with quantitative ultrasound index (QUI) as the outcome

	Model 0						Model 1					
	ACE model			AE model			ACE model			AE model		
	Estimate	95% CI	*p*	Estimate	95% CI	*p*	Estimate	95% CI	*p*	Estimate	95% CI	*p*
*Male*	NA			NA			0.38	0.04; 0.72	0.03	0.38	0.05; 0.72	0.025
*Age (/SD)*	NA			NA			− 0.36	− 0.52; − 0.19	< 0.0001	− 0.37	− 0.51; − 0.19	< 0.0001
*BMI (/SD)*	NA			NA			0.14	0.009; 0.27	0.036	0.15	0.004; 0.26	0.021
*Eversmoker*	NA			NA			− 0.19	− 0.43; 0.06	0.14	− 0.21	− 0.45; 0.04	0.096
*A*	42.9	22.4; 66.1	0.04	81.9	76.8; 86	< 0.0001	51.4	31.1; 71.3	0.022	77.2	70.7; 82.5	< 0.0001
*D*												
*C*	38.9	17; 66.5	0.085				25.2	5.8; 65	0.25			
*E*	18.2	12.9; 24.9	< 0.0001	18.1	13.1; 24.5	< 0.0001	23.4	16.9; 31.4	< 0.0001	22.8	16.7; 82.5	< 0.0001
*MZr*	0.84	0.67; 1	< 0.0001	0.84	0.67; 1	< 0.0001	0.75	0.56; 0.95	< 0.0001	0.75	0.56; 0.95	< 0.0001
*DZr*	0.6	0.32; 0.88	< 0.0001	0.6	0.32; 0.88	< 0.0001	0.54	0.24; 0.83	< 0.0001	0.54	0.24; 0.83	< 0.0001
− *2LL*	357.6			360			328.76			329.72		
*p*				0.12 (vs. ACE model)			< 0.0001 (vs. Model 0)			0.33 (vs. ACE model)		

*95% CI* 95% confidence interval, *A* additive genetic variance, *BMI* body mass index, *C* common or shared environmental variance, *D* dominant genetic variance, *DZr* intraclass correlation coefficient for dizygotic twins DZ twins, *E* unique environmental variance, *LL* log-likelihood, *MZr* intraclass correlation coefficient for monozygotic twins

*p*-values are given for the comparison of the − 2LL values between different models

*Model 0*: ACE-variance decomposition without adjustment

*Model 1*: Model 0 + age, sex, body mass index and ever smoker status (Model 1)

Both outcomes as well as age and BMI were normalized for these analyses; thus, 1 unit increase in TBS, QUI, age and BMI corresponds to one SD change (22 for QUI, 14.4 years for age and 5 kg/m^2^ for BMI)

## Discussion

### Short summary

In a study of altogether 82 Hungarian twin pairs in a classical twin design, we found a strong, over 70% genetic component of both TBS and QUI in unadjusted and adjusted models.

While the genetic component seemed to be additive in the unadjusted model of TBS, the adjusted model suggested a dominant genetic effect or even epistasis. It should be noted that half of all the variance was explained by independent variables (most strongly by age), leading to about a third (38.3%) of all variance decomposed to the genetic effect.

The unadjusted and adjusted models for QUI showed a more consistent result with over 75% of the variance decomposed to an additive genetic effect. The independent variables explained a quarter of the total variance leaving more than half of the variance for the additive genetic effect.

## Results in context

### TBS results in the context of the literature

To the best of our knowledge, this is the first study investigating the heritability of TBS in a classical twin design. Given this, it is difficult to put the findings of the present study in context of the literature. However, there is a family-based study that estimated the heritability of TBS in a south Asian population of similar age and sex distribution to our study. In general, our participants had higher BMD, and TBS, as well as a higher BMI compared to Vietnamese participants, probably reflecting the differences in socioeconomic status and ethnic admixture of the two populations. The observed role of the independent determinants was similar in the two studies; a large proportion of the observed variance in TBS was explained by age, sex, BMI and ever smoker status although the proportion of explained variance was almost two times higher in our study (48.0 vs. 28%). Similarly, the heritability estimates were also higher in our sample compared to the Vietnamese study (74 vs. 51% in unadjusted models). Furthermore, while the heritability in the family study was well described by the additive genetic model, our study supports a dominant genetic effect or even epistasis that means the gene by gene or gene by environment interactions could play an important role in the genetic architecture of TBS. These findings suggest that some assumptions of the ACE decomposition may not hold in our population (genes, gene and environment are not independent, EEA is not true) [26]. According to the literature, a higher heritability estimate is expected in twin compared to family (sibling) studies if the assumptions are broken [33].

Given that trabecular bone score is derived from the same lumbar spine DXA scan as the BMD, it seems reasonable to compare the heritability of lumbar BMD to TBS [34]. TBS is a grey-level textural measurement based on two-dimensional (2D) projection images obtained during a DXA scan of the bone 3D structure. However, the grey-scale variogram-based TBS correlates with the trabecular organization of the cancellous bone independently of the total amount of osseous tissue [35]. Furthermore, TBS strongly correlates with the number of trabeculae and their connectivity, and inversely with the space between trabeculae. A low TBS value indicates worse bone structure, whereas a high TBS value is considered better bone structure [34].

Previous twin and family studies estimate that the genetic contribution to BMD is about 60–90%. Heritability of BMD was frequently found to differ between the specific sites of measurement [22, 25, 36–41]. Similarly to our heritability findings on TBS, several of the twin studies found that the common environmental component was negligible and that the ratio of the MZ to DZ correlations of lumbar BMD was over 2, and thus a dominant genetic effect could be a better description of the findings than the ACE model although due to the low number of participants there was insufficient statistical power to prove this [19–22, 25]. Indeed our previous analysis is also compatible with this hypothesis [15]. It is also evident from the literature that measured environmental factors (such as age, body composition) are important independent predictors of BMD [17, 18, 20].

### QUI results in the context of the literature

Similarly to TBS, there is only limited data on the heritability of QUI in the current literature. We found no studies with the classical twin design and two family-based studies with one reporting a strong heritability (70–72%) [14], the other moderate heritability (48%) [42]. While both studies reported on mostly Caucasian populations, there is a large difference in the age of the included populations with mostly middle aged people in the Fels Longitudinal Study vs older participants in the Framingham Osteoporosis Study suggesting that the heritability decreases in older ages [14, 42]. Our results of a 77.2% additive heritability in middle aged twins are in line with the Fels Longitudinal Study findings [14]. Furthermore, although we only used a limited set of covariates (age, sex, BMI, ever smoker status) in our analysis compared to the other two studies, the variance explained by these variants seemed to be substantially larger in our compared to the other studies (26 vs 6–10 and 3–15%) [14, 42].

Our study extends previous findings in two notable ways. First, our data suggest an additive genetic component in the heritability estimates. Second, according to our data, the role of shared environment (in addition to age and BMI) is not a significant determinant of QUI.

Calcaneal QUS-derived variables (such as QUI) provide non-invasive measures that reflect both bone mass and quality. QUI well correlates with densitometric BMD [43, 44] and QUS measurements predict fractures [45–47], as well as other conditions such as skeletal integrity or trabecular bone structure at peripheral sites [48, 49]. Given that QUI is a linear combination of SOS and BUA and these measures (according to in vitro studies) reflect elasticity and density, as well as bone mass and microstructure [49–51], the heritability of SOS and BUA could further our understanding of the heritability of QUI.

Heritability estimates for SOS and BUA range from 0.19 to 0.58 and 0.43 to 0.74 in twin and 0.45 to 0.73 and 0.48 to 0.59 in family studies, respectively [14, 16, 22, 42, 52, 53]. While these estimates are mostly in the same ballpark, the heritability estimate of 19% in a British twin study seems to be an outlier [53]. This is explained by the fact that this is the only twin study where the shared environment (C) component of the ACE model is statistically significant (49%) and thus it is retained in the final model. It is conceivable that (given that this study reports only unadjusted estimates) adjustment for different covariates (such as age, weight, BMI) would diminish the C component to non-significance. Another interesting observation relates to the effect of age/postmenopausal status on the heritability of these measures. While the heritability of BMD measures decrease with aging/after the menopause, no such finding was reported for QUS measures pointing to a larger role of inheritance in QUS compared to BMD measures in elderly populations [53].

### Implications

Osteoporosis is characterized by low bone mass and microarchitectural deterioration of bone tissue leading to decreased bone strength and an increased risk of low-energy fractures. However, bone mineral density is only one of the principal determinants of osteoporotic fracture risk and BMD alone does not accurately identify fracture risk; thus, other skeletal properties such as bone quality should also be considered when determining bone strength and fracture risk [54–56]. This is supported by the fact that older patients are much more susceptible to fractures at any given bone mineral density than are younger patients [57].

Osteoporosis shows a strong age association and is considered an aging disease. Its consequences—in addition to fractures—include the aging syndrome of frailty, chronic obstructive pulmonary disease (COPD) and decreased lung function, as well as morbidity, and mortality. Approximately 30% of all the patients who sustain a hip fracture lose the ability to live independently, and in the year immediately after their fracture up to 20–30% die [58]. Furthermore, the interrelatedness of bone and muscular aging is clearly captured by the geriatric syndrome of osteosarcopenia [59–61].

While both bone quantity and quality as well as fat mass and lean mass can be easily measured by a simple DXA scan, bone measures are rarely used in aging clocks [62]; however, bone measures show a strong age dependence and correlation with other aging measures [63, 64]. Furthermore, bone aging is determined by the same overall aging mechanisms as other body systems [65]. Although osteoporosis was not related to epigenetic clocks in peripheral blood, a Mendelian randomization study suggested that bone measures had an effect on biological age measured by epigenetic clocks [66–68]. Given the above, we think that bone measures could provide additional information to frequently used phenotype-based aging clocks.

## Strengths and limitations

The major strength of our study is that (to the best of our knowledge) it is the first study investigating the heritability of TBS and QUI in a classical twin design. Twins are uniquely matched for sex, age and multiple unmeasured confounding variables, which gives twin studies a unique edge over family studies in separating shared environmental effects from genetic effects. We used state of the art methodology to measure these bone measures. Although the sample size is modest, given the multilevel structure of the data, we had sufficient power to investigate all components of the ACE model and adjust for important co-variates. While our results mostly confirmatory on the additive genetic inheritance of QUI, we found a dominant genetic inheritance or even epistasis for TBS that suggests that the investigation of the genetic determinants of TBS should take into account gene by gene and gene by environment interactions. Our work complements our previous study [13] that described the heritability of quantitative bone measures with similar estimation for bone quality measures. With the selection of age, sex, BMI and smoking habit, we included in our multivariate model the most important determinants of TBS and QUI that explain 5–10% of the variability of these measure [69–71].

The results of this study should be interpreted in light of its limitations. The validity of the classical twin design is dependent on the validity of its assumptions. However, it is likely that the monozygotic twins have a more similar environment compared to dizygotic twins that could lead to an overestimation of the genetic heritability. As all twins were measured at the same time and only same-sex twins were included, we were unable to decompose the effect of age and sex in the ACE models. Given the fact that all participants were of Caucasian origin, the external validity of our findings is limited. The external validity is further limited by the fact that the included population represents a convenience sample that is prone to bias related to study participation. While our multivariate models took into account the most important determinants of both TBS and QUI, some important determinants (such as prior fracture, rheumatoid arthritis, COPD, alcohol use, calcium intake) were not available in our analysis and thus heritability estimates may not reflect the true genetic effects [69–71]. Given these limitations, our results are probably generalizable to high-income countries with mostly Caucasian populations but findings could be different in low-income countries where the contribution of insufficient diet is much more marked. Furthermore, our estimates for the shared environmental component (C) have wide confidence intervals suggesting limited statistical power of our analysis. Given the cross-sectional nature of our study, it is prone to several types of bias, including healthy survival and reverse causality. Furthermore, only a limited set of co-variants were considered in our adjusted models; thus, the role of unmeasured confounding (i.e. physical activity, vitamin D levels, postmenopausal status and medications affecting bone health) cannot be excluded.

## Conclusion

To conclude, this twin study found a strong genetic heritability of different bone properties (TBS and QUI) determined by DXA and QUS in unadjusted models. However, we also found that half of the variance of TBS was explained by age, sex and BMI of the participants. Furthermore, the adjusted model also suggested that the genetic component of TBS is dominant or even an epistasis could be present that could hinder the investigation of the genes determining TBS. In contrast, independent variables explained only a quarter of the variance of QUI and the additive heritability explained more than half of all the variance.

## Declarations

The study was conducted in accordance with the Declaration of Helsinki and approved by National Scientific and Ethics Committee (ETT TUKEB 189–4/2014).
